# Use of selective serotonin reuptake inhibitors and risk of re-operation due to post-surgical bleeding in breast cancer patients: a Danish population-based cohort study

**DOI:** 10.1186/1471-2482-10-3

**Published:** 2010-01-24

**Authors:** Rune Gärtner, Deirdre Cronin-Fenton, Heidi H Hundborg, Lars Pedersen, Timothy L Lash, Henrik Toft Sørensen, Niels Kroman

**Affiliations:** 1 Department of Breast Surgery, Rigshospitalet, University of Copenhagen, Blegdamsvej 9 Copenhagen Ø 2100, Denmark; 2 Department of Clinical Epidemiology, Aarhus University Hospital. Olof Palmes Alle 43-45, Aarhus N 8200, Denmark; 3 National Centre for Register-based Research, University of Aarhus, Aarhus, Denmark; 4 Department of Epidemiology, Boston University School of Public Health, 715 Albany St. Boston, MA 02118, USA

## Abstract

**Background:**

Selective serotonin reuptake inhibitors (SSRI) decrease platelet-function, which suggests that SSRI use may increase the risk of post-surgical bleeding. Few studies have investigated this potential association.

**Methods:**

We conducted a population-based study of the risk of re-operation due to post-surgical bleeding within two weeks of primary surgery among Danish women with primary breast cancer. Patients were categorised according to their use of SSRI: never users, current users (SSRI prescription within 30 days of initial breast cancer surgery), and former users (SSRI prescription more than 30 days before initial breast cancer surgery). We calculated the risk of re-operation due to post-surgical bleeding within 14 days of initial surgery, and the relative risk (RR) of re-operation comparing SSRI users with never users of SSRI adjusting for potential confounders.

**Results:**

389 of 14,464 women (2.7%) were re-operated. 1592 (11%) had a history of SSRI use. Risk of re-operation was 2.6% among never users, 7.0% among current SSRI users, and 2.7% among former users. Current users thus had an increased risk of re-operation due to post-operative bleeding (adjusted relative risk = 2.3; 95% confidence interval (CI) = 1.4, 3.9) compared with never users. There was no increased risk of re-operation associated with former use of SSRI (RR = 0.93, 95% CI = 0.66, 1.3).

**Conclusions:**

Current use of SSRI is associated with an increased risk of re-operation due to bleeding after surgery for breast cancer.

## Background

Selective Serotonin Reuptake Inhibitors (SSRI) decrease platelet serotonin storage and platelet-function in humans [1,2] and are associated with upper gastrointestinal bleeding [3,4]. This mechanism suggests that SSRI use may increase the risk of post-surgical bleeding, but data on peri-operative risk of bleeding associated with use of SSRI are few and contradicting [5,6]. One Danish study on coronary artery bypass surgery reported no association between the use of SSRI and an increased requirement for blood transfusion [5]. In contrast, a study on orthopaedic surgery found use of SSRI associated with a 3.7-fold increased risk of subsequent blood transfusion [6]. Severe peri-operative bleeding, defined as the need for blood transfusion following breast cancer surgery, is a rare event. Even so, major postoperative bleeding requiring re-operation occurs in about 4% of women operated on for breast cancer [7].

The prevalence of patients using SSRI is increasing. According to The Danish Medicines Agencys' data, usage has increased from 38.3 Defined Daily Doses/1000 inhabitants in 2004 to 50.3 in 2008 [8]. Additionally, breast cancer is the most common cancer among women, with surgery the primary treatment. Thus, an increased risk of post-surgical bleeding associated with SSRI use among breast cancer patients may have important clinical implications.

We therefore conducted a population-based study with prospectively collected administrative data to examine the association between use of SSRI and re-operation due to post-surgical bleeding in a large cohort of Danish women undergoing breast cancer surgery.

## Methods

### Study population

We conducted this population-based cohort study among residents of Northern Denmark, which has a total population of 1.7 million inhabitants. The Danish National Health Service provides tax-supported healthcare to all residents of the country and refunds part of patient expenditures on a wide range of prescribed drugs, including SSRI. A unique civil personal registration (CPR) number has been assigned to all Danish citizens since 1968 by the Danish Civil Registration System. This number encodes gender and date of birth [9], and facilitates accurate linkage between population-based registries.

All hospitalizations are registered to individual patients in the National Registry of Patients, which has covered all Danish hospitals since 1977. Data from the National Registry of Patients on the inhabitants of Northern Denmark have been merged into a research database at Aarhus University [10,11]. This database includes all non-psychiatric hospital admissions since 1977, and outpatient and emergency room data since 1994. Information is recorded in the National Registry of Patients immediately after discharge or outpatient visit and includes CPR number, dates of admission and discharge, and up to 20 diagnostic codes categorized by ICD code [12]. Using the National Registry of Patients, we identified 14,464 female patients who had a first diagnosis of breast cancer (ICD-10 codes C50.0-50.6, C50.8 & C50.9) from 1 January 1996 through 31 March 2007, the time period during which we could link to complete prescription history by the methods described below.

### Post-operative bleeding outcomes

Information on re-operation due to post-surgical bleeding within 14 days of primary breast cancer-directed surgery was retrieved from the National Registry of Patients in accordance with the Danish Classification of Surgical Procedures and Therapy (codes: KHWD00, KHWE00). All breast cancer patients were treated with either mastectomy (code KHAC) or breast-conserving surgery (code KHAB).

### SSRI prescription data

All pharmacies in Northern Denmark use computerised accounting systems connected with the National Health Service to record a customers' CPR number, type and quantity of medication dispensed (including tablet, package sizes, and number of days on prescription), and prescription data according to the drug's Anatomical Therapeutic Classification (ATC) (World Health Organization Collaborating Centre for Drug Statistics Methodology, 2001). Data on prescriptions for refundable drugs are transferred electronically to the National Health Services and to the research database at Aarhus University, with complete coverage in the region since 1998. In Denmark, anti-depressants are available only by prescription. We identified prescriptions for the following SSRI anti-depressants: Fluoxetine N06AB03, citalopram N06AB04, paroxetine N06AB05, sertraline N06AB06, fluvoxamine N06AB08, and escitalopram N06AB10. Patients were categorised according to their use of SSRI: never users, current users (prescription of SSRI within 30 days before initial breast cancer surgery), and former users (prescription of SSRI more than 30 days before initial breast cancer surgery).

### Data on potential confounders

To account for factors that may be associated with SSRI anti-depressant prescription and post-surgical bleeding, we obtained data on age at breast cancer surgery and on preoperative use of platelet inhibitors, vitamin K antagonists, oral anti-coagulants, non-steroidal anti-inflammatory drugs (NSAID) (non-aspirin NSAID, excluding selective Cox-2 inhibitors, as these do not impair platelet function [13]) non-SSRI anti-depressants (tri-cyclic anti-depressants (TCA), tetracyclical anti-depressants (TeCA), and other anti-depressants) (see Appendix for specific ATC codes). We also obtained information on specific precedent comorbidities including liver disease, uraemia, other cancers, renal diseases, autoimmune diseases, thrombocytopenia, and vasculitis; all of which can cause bleeding.

### Statistical analyses

Follow-up began at the date of primary breast cancer surgery and continued until fourteen days after the operation or 1 April 2007. We analysed the data first by tabulating contingency tables for the main study variables, from which we calculated the risk of re-operation due to post-surgical bleeding according to use of SSRI. We computed the crude risk difference and risk ratio with their 95% confidence intervals associating SSRI prescription with post-operative bleeding. We then stratified the contingency tables according to each of the possible confounding variables to examine the strength of confounding. We also fitted multiple logistic regression models to the data to compute the odds ratio and associated 95% confidence intervals that controlled for confounding by identified confounders. Given that re-operation for post-surgical bleeding was rare in all combinations of the independent variables, these adjusted odds ratios provide an estimate of the adjusted risk ratios (aRR). In the final model we included variables that changed the exposure odds ratio by 10 percent [14]. All data analyses were performed using Stata 9.0.

### Ethics

Studies based on registry data do not require formal ethical approval under Danish law. However, handling of data was approved by the Danish Data-protection Agency.

## Results

Characteristics of the cohort of 14,464 breast cancer patients according to SSRI prescription are presented in Table 1. Approximately 65% of patients were over 50 years old at initial breast cancer surgery, 60% underwent breast-conserving surgery, and 40% underwent mastectomy as their primary breast cancer-directed surgery. Slightly more women who had ever used SSRI compared with those who had not used SSRI had also used other prescription drugs, including NSAID, aspirin, corticosteroids, vitamin K antagonists and platelet inhibitors. Women with a history of SSRI prescription had at least one of the selected precedent comorbid diseases more often than those who had not used SSRI (12% versus 9%).

**Table 1:** Characteristics of 14,464 breast cancer patients^# ^according to SSRI prescription.

Characteristics	No SSRI Prescription N (%)	Ever SSRI Prescription N (%)	Total Population N (%)
**Age Group**			
<40	2010 (16)	147 (9)	2157 (15)
40-49	2669 (21)	311 (20)	2980 (21)
50-59	3117 (24)	373 (23)	3490 (24)
60-69	2441 (19)	313 (20)	2754 (19)
70-79	1699 (13)	258 (16)	1957 (14)
> = 80	936 (7)	190 (12)	1126 (8)

**Temporality of Anti-depressant (including SSRI, TCA, TeCA, others) Prescription**			
Never	11857 (92)	0	11857 (82)
Current	266 (2)	411 (26)	677 (5)
Former	749 (6)	1181 (74)	1930 (13)

**Primary operation type**			
Breast-conserving surgery	7799 (61)	936 (59)	8735 (60)
Mastectomy	5073 (39)	656 (41)	5719 (40)

**Anti-depressant prescription**			
Never	11857 (92)	0	11857 (82)
SSRI	0	1592 (100)	1592 (11)
Tricyclic anti-depressants	526 (4)	0	526 (4)
Tetracyclic anti-depressants	131 (1)	0	131 (1)
Other	358 (3)	0	358 (3)

**Oral Anti-coagulants prescription**			
No	12860 (99.9)	1589 (99.8)	14449 (99.9)
Yes	12 (0.1)	3 (0.2)	15 (<0.1)

**NSAIDs prescription**			
No	6134 (48)	493 (31)	6627 (46)
Yes	6738 (52)	1099 (69)	7837 (54)

**Aspirin prescription**			
No	12164 (95)	1423 (89)	13587 (94)
Yes	708 (6)	169 (11)	877 (6)

**Corticosteroid prescription**			
No	10969 (85)	1194 (75)	12163 (84)
Yes	1903 (15)	398 (25)	2301 (16)

**Vitamin K Antagonists Prescription**			
No	12599 (98)	1529 (96)	14128 (98)
Yes	273 (2)	63 (4)	336 (2)

**Platelet Inhibitors Prescription**			
None	11924 (93)	1341 (84)	13265 (92)
Yes	948 (7)	251 (16)	1199 (8)

Comorbid diseases*			
None	11764 (91)	1396 (88)	13160 (91)
Yes	1108 (9)	196 (12)	1304 (9)

**Comorbid Diseases**			
Liver disease	72 (0.6)	23 (1.4)	95 (0.7)
Renal disease	89 (0.7)	27 (2)	116 (0.8)
Cancer	951 (7)	141 (9)	1092 (8)
Thrombocytopoenia	7 (0.05)	1 (0.06)	8 (0.06)
Auto-immune disease	3 (0.02)	3 (0.02)	6 (0.04)
Vascular disease	12 (0.1)	3 (0.2)	15 (<1)

^#^Women diagnosed in Northern Denmark between 1996 and 2007 with complete prescription coverage since 1999.

*Comorbid diseases includes liver disease, renal disease, uremia, cancer, thrombocytopoenia, auto-immunities, vasculitis

SSRI = Selective serotonin reuptake inhibitors, TCA = Tricyclic anti-depressants, TeCA = Tetracyclic anti-depressants

Risk of re-operation due to post-surgical bleeding after breast cancer surgery is presented in Table 2. In all, 389 of 14,464 women (risk = 2.7%) in the breast cancer cohort underwent a re-operation due to post-surgical bleeding within fourteen days following initial breast cancer surgery. 338 of 12,872 never users of SSRI underwent a re-operation (risk = 2.6%), 14 of 201 current users of SSRI underwent a re-operation [risk = 7.0%, risk difference = 4.3% (95% CI 0.8%, 7.9%), risk ratio = 2.7 (95% CI 1.6, 4.4)], and 37 of 1391 former users of SSRI underwent a re-operation [risk = 2.7%, risk difference = 0.03% (95% CI -0.9%, 0.9%), risk ratio = 1.0 (95% CI 0.72, 1.4)].

**Table 2:** Risk of re-operation due to post-surgical bleeding after breast cancer surgery according to SSRI prescription among 14,464 women^#^.

	Re-operation/Cohort members	Risk (%)	Crude RR (95%CI)	Adjusted RR* (95%CI)
**SSRI Prescription**				
Never	338/12872	2.6	1.0	1.0
Ever	51/1592	3.2	1.2 (0.91, 1.7)	1.1(0.83, 1.5)

**Temporality^ of SSRI prescription**				
Never	338/12872	2.6	1.0	1.0
Current	14/201	7.0	2.7 (1.6, 4.4)	2.3 (1.4, 3.9)
Former	37/1391	2.7	1.0 (0.72, 1.4)	0.93 (0.66, 1.3)

Risk of re-operation occurring in the first fourteen days after surgery was estimated in never, ever, current and former users.

Current users were defined as women with a prescription of SSRI within 30 days before initial breast cancer surgery.

Former users were defined as women with a prescription of SSRI more than 30 days before initial breast cancer surgery.

^# ^Women diagnosed in Northern Denmark between 1996 and 2007 with complete prescription coverage since 1999.

^Temporality of current use of SSRI was defined as prescriptions within 30 days prior to admission for surgery.

*Adjusted for age

SSRI = Selective serotonin reuptake inhibitors, RR = risk ratio, CI = 95% confidence interval

The elevated risk of re-operation among current users of SSRI remained after adjustment for age (aRR = 2.3, 95%CI 1.4, 3.9) when compared with never users. Similarly, the near-null association between former use of SSRIs and re-operation due to post-surgical bleeding remained after adjustment for age (aRR = 0.93, 95%CI 0.66, 1.3). Further adjustment for other medications or the selected potential confounding comorbid diseases did not substantially affect the risk ratio estimates of association.

## Discussion

We hypothesized that current use of SSRI would increase the risk of re-operation after breast cancer surgery due to postoperative bleeding, and the results of this investigation were consistent with that hypothesis. While current users of SSRI had almost tripled the risk of re-operation due to postoperative bleeding, the absolute change in risk was only from 2.7% to 7.0%.

The present study is population-based with complete prescription and follow-up data. Recall-bias was eliminated by use of prescription records generated before the initial breast cancer surgery date and all data were obtained prospectively from population-based registries that have a completeness approaching 100% [15]. This information was linked together by the unique CPR number [9] that has been used productively for research housed in the Danish registries for 15 years [16,17].

Our comparison of post-surgical bleeding risk in SSRI users versus never SSRI users may raise a concern about non-comparability of the baseline risk of post-surgical bleeding related to indications for SSRI use. For example, patients with a history of depression, which is the primary indication for SSRI use, may tend to have a less healthy lifestyle than patients without a history of depression with respect to tobacco use, diet, alcohol use, and exercise [18,19]. Depressed patients may also more often use pain medications, such as NSAID, which are known to impair platelet aggregation and thus increase risk of bleeding. This non-comparability of baseline risks would lead to an overestimation of the effect of SSRI use on the risk of re-operation. We controlled confounding by the most frequently used drugs and comorbid diseases related to coagulation and bleeding, and this control had little impact on the estimates of association. However, we were unable to control for NSAIDs bought over the counter or alcohol consumption, as we have no reliable registry data on these habits.

A limitation of the current study is that it includes no information on SSRI prescription compliance. For example, women defined as current users may not have taken the medication up to the day of surgery. Our information on SSRI use relied on registration of dispensed prescriptions and the Danish health care system covers only a proportion of the costs of prescribed medicine. Dispensed prescriptions for which patients must pay part of the cost are likely to be actually used. Furthermore, women on anti-depressant medication with the additional distress of a newly diagnosed breast cancer would be unlikely to stop their anti-depressant medication. Earlier studies on orthopaedic surgery and coronary artery bypass surgery [5,6], have used the requirement of blood transfusion as a proxy for peri-operative bleeding when investigating its association with SSRI use. We used re-operation due to post surgical bleeding, because peri-operative bleeding in the case of breast cancer surgery rarely results in blood transfusion. In addition, registration of re-operation due to post surgical bleeding in the Danish National Registry of Patients is virtually complete [20-22].

The overall risk of re-operation due to postoperative bleeding in breast cancer patients observed in our study was of the same magnitude as that observed in a small single centre cohort (0.8-4.1%) [7], which adds further support to the validity of our outcome measure.

The clinical relevance of SSRI in relation to bleeding has been discussed for almost two decades [1,2,23]. Breast cancer surgery is a soft tissue surface-surgery often characterised by extensive dissection, which increases the risk of postoperative bleeding compared with many other surgical soft tissue procedures. As we found an increased risk of re-operation due to post-surgical bleeding associated with current use of SSRI, our findings may also be relevant to other soft-tissue surgical procedures of similar character where bleeding might have more fatal consequences.

Our results show overall low risk of re-operation due to post-surgical bleeding among breast cancer patients regardless of whether they are current, former, or never users of SSRI medications. The consequence of stopping SSRI medication used to treat depression in women undergoing treatment for breast cancer may be overwhelming, and may even delay any subsequent cancer-directed treatment. Thus, the potential consequences of stopping the treatment -- taken together with the low overall risk -- suggests that physicians and patients should discuss the risks and benefits of discontinuing treatment with SSRI before breast cancer surgery.

## Conclusion

Use of SSRI is associated with an increased risk of re-operation due to bleeding after surgery for breast cancer, from about 3% to about 7%. However, the magnitude of the risk difference does not merit pausing antidepressive medicine in psychologically vulnerable patients without a complete discussion of the risks and benefits.

## Abbreviations

(SSRI): Selective serotonin reuptake inhibitors; (CPR): Civil Personal Registration; (ICD): International Statistical Classification of Diseases and Related Health Problems; (NSAIDs): non-steroidal anti-inflammatory drugs; (TCAs): tri-cyclic anti-depressants; (TeCA): tetracyclical anti-depressants; (ATC): Anatomical Therapeutic Chemical.

## Competing interests

The authors declare that they have no competing interests.

## Authors' contributions

RG put forward the hypothesis; made substantial contributions to conception and design and interpretation of the data; and drafted the paper: abstract, introduction, and discussion. DCF was involved in study conception and design; performed the statistical analysis, data interpretation and conclusions; drafted the methods and results section; reviewed and edited the paper. HHH & LP provided the datasets and statistical analysis and support. TLL was involved in revising the manuscript critically for important intellectual content and consistency regarding analysis, interpretation and conclusions. HTS was involved in study conception, design and data collection and revised the manuscript critically for important intellectual content and consistency regarding analysis, interpretation and conclusions. NK supported the development of the hypothesis, helped to draft the paper and was involved in the clinical interpretation of the results and revision of the manuscript for important intellectual content.

All authors read and approved the final manuscript.

## APPENDIX

ATC codes for potential confounding drugs:

**Platelet inhibitors**: (low-dose aspirin B01AC06 and N02BA01 in tablet sizes of 75, 100 or 150 mg;

**Vitamin K antagonists**: (heparin B01AB01; warfarin B01AA03; phenprocoumon B01AA04), dipyridamol B01AC07; clopidogrel B01AC04);

**Oral anti-coagulants**: (B01AB02, B01AB04, B01AB05, B01AB08, B01AB10, B01AC06, B01AC09, B01AC13, B01AC14, B01AC16, B01AC17, B01AC30, B01AD01, B01AD02, B01AD04, B01AD07, B01AD10, B01AD11, B01AE04, B01AE05, B01AX03, B01AX05);

**NSAIDs**: (non-aspirin NSAIDs M01A*, excluding selective Cox-2 inhibitors);

**Anti-depressants**: Tri-cyclic anti-depressants (TCAs): Desipramine N06AA01; imipramin N06AA02; imipramine oxide N06AA03; clomipramin N06AA04; opipramol N06AA05; trimipramine N06AA06; lofepramine N06AA07; amitriptylin N06AA09; nortriptylin N06AA10; protriptyline N06AA11; doxepin N06AA12; dosulepin N06AA16; amoxapine N06AA17; Tetracyclical anti-depressants (TeCA): Maprotilin N06AA21, Mianserin N06AX03; Other anti-depressants: Duloxetin N06AX21, venlafaxin N06AX16, Mirtazapin N06AX11, Reboxetin N06AX18, Isocarboxazid N06AF01, Moclobemid N06AG02.

## Pre-publication history

The pre-publication history for this paper can be accessed here:

http://www.biomedcentral.com/1471-2482/10/3/prepub
